# Aspirin and immunotherapy: a Faustian bargain?

**DOI:** 10.1172/JCI169598

**Published:** 2023-05-01

**Authors:** Eric A. Goethe, Amy B. Heimberger, Ganesh Rao

**Affiliations:** 1 Baylor College of Medicine Department of Neurosurgery, Houston, Texas, USA.; 2 Lurie Comprehensive Cancer Center, Feinberg School of Medicine, Northwestern University, Chicago, Illinois, USA.

## Abstract

Fibrinogen-like protein 1 (FGL1) has been associated with improved survival in hepatocellular carcinoma (HCC). However, recent evidence suggests that FGL1 may bind to surface receptors on lymphocytes and induce immune senescence. In this issue of the *JCI*, Lin and co-authors show that FGL1 may be acetylated by aspirin and targeted for degradation, which is associated with increased antitumor immunity and improved survival. Similar findings were obtained with inhibitors of sirtuin 2 (SIRT2), a histone deacetylase. These findings expand our current understanding of the role of FGL1 in cancer and provide an impetus for the evaluation of alternative immunotherapy combinations in HCC.

## The role of FGL1 in hepatocellular carcinoma

Cancer evades detection by the immune system by utilizing various modulators, such as programmed cell death protein 1 (PD-1) and lymphocyte-activating gene 3 (LAG-3) (1). LAG-3 is expressed on the surface of T cells, and its activation suppresses T cell antigen–mediated activation, signaling, and proliferation (2, 3). Fibrinogen-like protein 1 (FGL1) has been identified as a ligand of LAG-3 and is implicated in several malignancies (1, 2). Antibody-mediated knockdown of FGL1 expression has been shown to increase antigen-mediated T cell activation. However, the role of FGL1 in cancer is likely contextual and lineage dependent, since its inhibition promotes growth in lung cancer cells but exerts antitumoral effects in gastric and colon cancer (2, 3).

**Figure 1:**
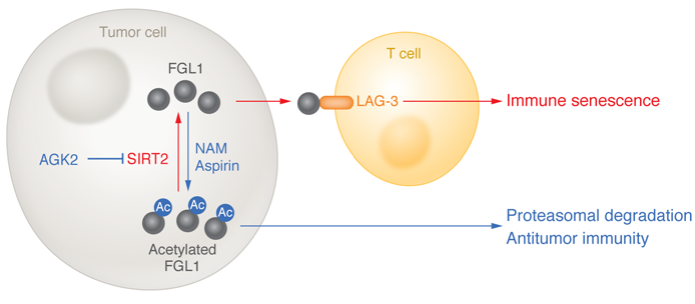
Acetylation of FGL1 has a regulatory role in HCC antitumor immunity. Acetylation of FGL1 at Lys-98 reduces the levels of FGL1 in HCC cells due to increased proteasomal degradation. The NAD^+^-dependent deacetylase SIRT2 decreases FGL1 acetylation, which facilitates interaction between LAG-3 to promote evasion from immune surveillance. In contrast, compounds that increase acetylated FGL1, including the SIRT2 inhibitor AGK2, NAM, and aspirin, increase FGL1 degradation and antitumor immunity via T cell–mediated killing.

In this issue of the *JCI*, Lin and colleagues (4) evaluated the role of FGL1 in hepatocellular carcinoma (HCC). Nicotinamide (NAM) treatment resulted in the acetylation of FGL1 at Lys-98, which reduced the levels of FGL1 in HCC cell lines due to increased proteasomal degradation. Expression of sirtuin 2 (SIRT2), a NAD^+^-dependent deacetylase, triggered a decrease in FGL1 acetylation, while inhibition of SIRT2, by either the specific inhibitor acetylglutamate kinase 2 (AGK2) or an siRNA, resulted in increased acetylation and decreased levels of FGL1. Similarly, aspirin was shown in vitro to acetylate FGL1, leading to its proteasomal degradation (Figure 1). AGK2 was associated with increased T cell–mediated killing of HCC cells in vitro and prolonged survival, alone, and in synergy with PD-L1 inhibition, in mice. Likewise, aspirin was associated with enhanced T cell destruction of HCC cells. The authors also demonstrated improved survival in mice with HCC that were treated with aspirin and a PD-L1 inhibitor. AGK2 and aspirin were equally effective with LAG-3 inhibition at suppressing tumor growth (4).

The Lin et al. study mechanistically shows that FGL1 can be regulated by acetylation at K98 via AGK2 or aspirin treatment in vitro and excluded other acetylation sites or mechanisms of degradation. While the authors provide mechanistic support for this result, one potential weakness was the lack of assessment for FGL1 acetylation in the in vivo experiments, or in assays of T cell–mediated tumor killing, raising the possibility that antitumor immunity results from some unmeasured effect. However, the same agents shown to acetylate FGL1 in vitro were used for these experiments, and the in vivo experiments demonstrated reduced FGL1 expression by immunohistochemistry (4).

### FGL family in cancer.

Both FGL1 and FGL2 share the duality of modulating both tumorigenesis and immune responses. FGL1 was originally cloned from HCC, and its expression is elevated in melanoma, colorectal, prostate, lung, gastric, and breast cancers based on BioGPS tissue microarrays and The Cancer Genome Atlas (TCGA) database (5). FGL1 has been identified as a major ligand for LAG-3. FGL1 can suppress Akt signaling, but FGL1 can be suppressed by IL-6–mediated JAK2/STAT3 signal activation (6). FGL2 was previously shown by our group to be involved in the malignant transformation of low- to high-grade gliomas and is expressed in other cancers, such as HCC, prostate, B cell lymphoma, colorectal, and lung cancers (7). Knockdown or silencing of FGL2 expression has also been shown to inhibit prostate and lung cancer growth in preclinical models (8–10). FGL2 can drive tumorigenesis through ERK1/-2 and the MAPK kinase pathways. The immune-modulatory functions of FGL2 relative to FGL1 are more extensively described, in general and in the context of cancer. During viral infection, FGL2^–/–^ mice possess a higher frequency of DCs expressing CD80, CD86, and MHC; they also show decreased PD-1 expression on T cells and increased B and T cell effector responses (11). We have shown through a series of studies of preclinical glioma that FGL2 increases the expression of PD-1 and the frequency of tumor-supportive macrophages and Tregs, suppresses the development of CD103^+^ DCs, and blocks the recruitment of tumor-specific, brain-resident memory T cells (7, 12, 13). In other cancers, FGL2 expression was associated with better prognostic outcomes and increased lung cancer infiltration of immune cells, such as CD8^+^ T cells, macrophages, B cells, and DCs (14). As such, both FGL1 and FGL2 have been widely reported to share both pro- and antitumor functions.

### The role of FGL1 in tumorigenesis.

The results of Lin et al. indicate that FGL1 expression is a negative prognosticator in HCC, as demonstrated by the association between FGL1 inhibition, antitumor immunity, and survival (4). Notably, these results contrast with prior findings in which downregulation of FGL1 in HCC increases the rates of tumor formation (15–17), and is a positive prognosticator (15). The discrepancy between the present study and others may be a function of analyzing the different ways in which FGL1 influences the development and progression of HCC. FGL1 loss is associated with tumor dedifferentiation and increased Akt pathway signaling in HCC and is probably an effect independent of antitumor immunity (17). Additionally, multiple other tumor suppressor loci are near FGL1, suggesting that loss of the gene (i.e., via chromosomal damage) may be linked to other tumor suppressor genes (17). Thus, the differences between studies may be a function of unaccounted coassociated tumor suppressor genes and the balance of antitumor and anti-immune responses in various models. The Lin et al. study (4) emphasizes the complexity of outcome biomarkers that play different and opposing roles in tumorigenesis. Loss of FGL1 may disinhibit HCC proliferation yet contribute to an antitumor immune response. As such, the role of FGL1 in HCC is less clear than previously presumed. Nonetheless, an alternative understanding of previously established tumor markers can drive further research and treatment development.

## FGL1 and antitumor immunity

Lin et al. demonstrate that acetylation-mediated FGL1 inhibition via AGK2 was associated with increased T cell killing of HCC cells in vitro and in vivo (4). Combination therapy with a PD-L1 inhibitor and AGK2 resulted in further increases in antitumor immunity. An association between FGL1 inhibition and antitumor immune responses has been reported in other studies (1, 3). Wang et al. (3) implanted FGL1-knockout mice with colon cancer cells and found diminished tumor growth compared with controls. Furthermore, depletion of B and T cells mitigated the antitumor effects of FGL1 and LAG-3 inhibition, suggesting that FGL1 contributes to tumor growth by inhibiting antitumor immunity (3). Huang et al. (1) found that FGL1 silencing was associated with increased intratumoral T cell activity in a murine model of squamous cell carcinoma. Thus, it is unsurprising that the combination of FGL1 and PD-1 inhibition, which would be expected to bolster the antitumor immune response, resulted in further benefits in tumor control and survival in the Lin et al. study (4). High FGL1 expression is associated with low responsiveness to anti–PD-1 therapy in lung cancer (18), and these results present a unique opportunity to validate similar combination regimens in HCC.

## Aspirin as an anticancer drug

Goethe’s Faust forfeits his soul for the temptations of unlimited knowledge and earthly pleasure. While it may be tempting to label aspirin an anticancer drug on the basis of the results of Lin et al. (4), doing so may represent a similar exchange, in which one sacrifices a comprehensive and rigorous evaluation of aspirin’s role in cancer in exchange for an easy and immediate result — a choice that could have similarly damaging consequences. In 2007, aspirin was reported to reduce the risk of colorectal cancer in a systematic review, prompting a flurry of studies examining its role in the prevention of this and other malignancies (19). However, a recent umbrella review of the role of aspirin in cancer prevention raised several concerns about the validity of such studies, primarily due to a lack of stringent significance criteria and substantial heterogeneity (19). Only one of these studies examined the use of aspirin in HCC, demonstrating no preventive role of the drug (20). One study cited by Lin and authors suggests a dose-dependent reduction in HCC risk with aspirin use, but the benefits were only apparent after five years of use, a time period extending beyond the expected survival of patients with HCC (21, 22). Aspirin has many anticancer properties, such as inhibition of proliferation, promotion of apoptosis, and suppression of stemness, thus, it is unlikely that one can tease apart the underlying mechanism as being attributed exclusively to the modulation of FGL1 (23). Notably, aspirin has also been shown to suppress PD-L1 expression; however, the use of aspirin in the clinical setting of melanoma was not shown to impact the efficacy of anti–PD-1 (24–26). Thus, while the results of Lin et al. expand a relatively narrow body of evidence by suggesting a therapeutic strategy for targeting HCC using a common drug, the results should be interpreted with caution, considering the above concerns and the possible coagulopathy accompanying HCC that may be exacerbated with aspirin use (27).

## Acetylation-mediated cancer therapy

The authors also used SIRT2 inhibition to induce acetylation of FGL1 to facilitate its proteasome-mediated degradation (4). Targeting acetylation pathways has emerged as a potential therapeutic strategy in cancer (28, 29). Histone deacetylases (HDACs) like SIRT2 can remove acetyl groups from both histone and nonhistone proteins, thereby sensitizing cells to chemo- and radiotherapy by hindering double-stranded DNA break repair (28). By demonstrating an association between SIRT2 inhibition, FGL1 acetylation, and antitumor immunity, the Lin et al. results indicate that HDAC inhibitors may have multiple effects in cancer treatment. One important consideration is whether tumor cell death and improved survival in this study were caused by DNA damage incurred by HDAC inhibition rather than via antitumor immunity — indeed, the authors did not assess DNA damage (4). However, the authors did demonstrate enhanced tumor invasion by immune cells in response to FGL1 acetylation (4), and past research has shown FGL1–LAG-3 interaction (1, 3). Thus, it is likely that antitumor immunity at least plays a role in these findings. Further study will be needed to fully delineate the relative contributions of each of these mechanisms to observed preclinical therapeutic activity.

## Conclusion

While FGL1 may behave as a tumor suppressor in HCC, Lin and authors have illuminated a role of FGL1 by highlighting its contribution to antitumor immunity in HCC. Notably, its inhibition was associated with an increased antitumor immune response with PD-1 inhibitors. These data provide impetus for clinical evaluation of immunotherapy regimens, but the utility of aspirin should be interpreted with caution, given the largely negative results regarding its efficacy against cancer.

## Version 1 05/01/2023

Electronic publication
